# Tuberculosis reactivation at ileum following immune checkpoint inhibition with pembrolizumab for metastatic nasopharyngeal carcinoma: a case report

**DOI:** 10.1186/s12879-021-06845-7

**Published:** 2021-11-10

**Authors:** Kin-Sang Lau, Ben Man-Fei Cheung, Ka-On Lam, Sum-Yin Chan, Ka-Ming Lam, Chun-Fai Yeung, Ivan Fan-Ngai Hung, Dora Lai-Wan Kwong, Chi-Chung Tong, To-Wai Leung, Mai-Yee Luk, Anne Wing-Mui Lee, Kwok-Keung Yuen, Victor Ho-Fun Lee

**Affiliations:** 1 Department of Clinical Oncology, LKS Faculty of Medicine, The University of Hong Kong, Hong Kong, China; 2 Department of Pathology, Queen Mary Hospital, Hong Kong, China; 3 Department of Medicine, LKS Faculty of Medicine, The University of Hong Kong, Hong Kong, China

**Keywords:** Nasopharyngeal carcinoma, Immune checkpoint inhibitors, Tuberculosis reactivation, IGRA

## Abstract

**Background:**

Tuberculosis (TB) reactivation has been increasingly identified following immune checkpoint inhibitor (ICI) therapy for cancer patients. However there has been no report on TB reactivation in the gastrointestinal tract. In the report, we describe a patient who developed TB ileitis after pembrolizumab for her metastatic nasopharyngeal carcinoma (NPC). Rechallenge with pembrolizumab after its temporary interruption together with anti-TB therapy produced continuous tumor response but without further TB reactivation.

**Case presentation:**

A 29-year-old lady with metastatic NPC involving the cervical nodes, lungs and bones started pembrolizumab after failure to multiple lines of chemotherapy. She complained of sudden onset of abdominal pain, vomiting and bloody diarrhea with mucus 21 months after pembrolizumab. Colonoscopy revealed terminal ileitis with multiple caseating granulomas with Langerhan cells. Serum interferon gamma release assay was strongly positive. She was treated with anti-TB medication and was later rechallenged with pembrolizumab for her progressive lung metastases without further TB relapse while her lung metastases were brought under control again.

**Conclusion:**

To date, this is the first gastrointestinal TB reactivation after ICI therapy for cancer. Guidelines to screen for TB before initiation of ICIs in endemic areas should be established.

## Background

Immune checkpoint inhibitors (ICI) against programmed death-(ligand)1 (PD-(L)1) and cytotoxic T-lymphocyte-associated antigen 4 have gained increasing popularity for various malignancies [1]. Despite improvement in progressive-free survival and overall survival particularly in melanoma and lung cancer, immune-related adverse events (irAE) have also been increasingly reported [2]. There has a dozen of case reports of tuberculosis (TB) reactivation after ICI therapy so far, including two patients who developed pulmonary TB reactivation during ICI therapy for their metastatic nasopharyngeal carcinoma (NPC) [3–15]. However, the incidence of TB reactivation after ICI therapy is western countries is exceedingly rare [16]. Here, we report a patient with metastatic NPC who developed TB ileitis during pembrolizumab therapy and was subsequently treated with a full course of anti-TB antibiotics with temporary ICI interruption. She was successfully re-challenged with pembrolizumab during anti-TB treatment, with her tumours still in continuous response. Various strategies on identifying latent TB infection before initiation of anti-TB prophylaxis are proposed.

## Case presentation

**Fig. 1:**
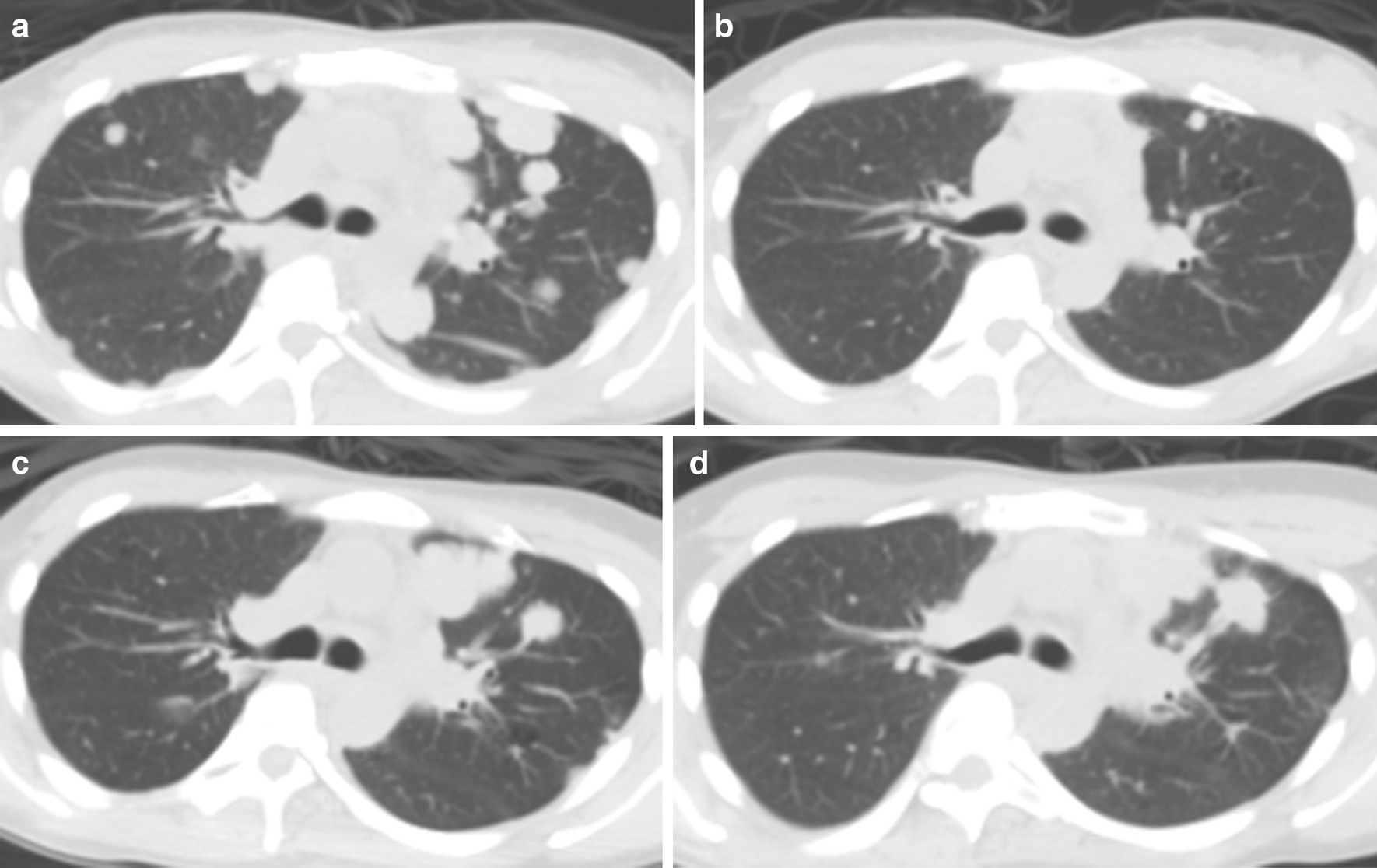
Serial positron emission tomography with integrated computed tomography images showing response to lung metastases before and after pembrolizumzb treatment, interruption and rechallenge during and after anti-tuberculosis (TB) therapy. **a** Multiple bilateral lung metastases before pembrolizumab treatment. **b** Marked reduction in size and number of lung metastases after pembrolizumab treatment. **c** Enlarging lung metastases after pembrolizumab interruption due to TB reactivation. **d** Tumour shrinkage after re-challenge with pembrolizumab

**Fig. 2:**
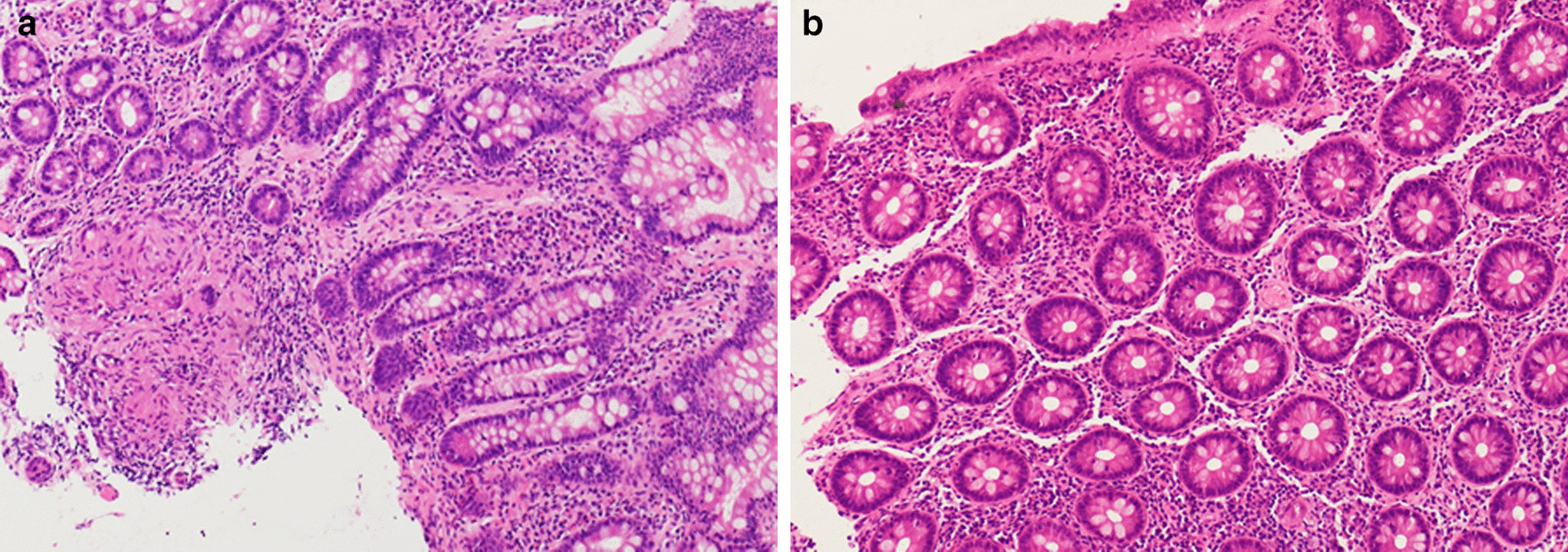
Ileal biopsies of our patient who developed tuberculosis (TB) reactivation after pembrolizumab and resolution after anti-TB therapy (image resolution 2880 × 2048 without additonal processing made). **a** TB ileitis with small well-formed caseating granulomas after pembrolizumab. **b** Resolution of TB ileitis after initiation of anti-TB medication and pembrolizumb interruption

A 29-year-old lady was diagnosed with metastatic NPC with cervical nodal, lung and bone metastases in December 2014. Her disease progressed despite multiple lines of chemotherapy including gemcitabine and cisplatin (3 cycles from December 2014 to March 2015)cisplatin plus 5-fluorouracil (1 cycle in April 2015) which was changed to docetaxel plus cisplatin because of 5-fluorouracil allergy (3 cycles from April 2015 to June 2015), gemcitabine and carboplatin (6 cycles from November 2015 to March 2016) capecitabine (6 cycles from April 2016 to August 2016), and metronomic cyclophosphamide (from August 2016 to September 2016). Radical chemoradiation with cisplatin was given to her progressive neck nodes in July 2015. Her lung metastases later further progressed resulting in mild dyspnea and her plasma Epstein-Barr virus (EBV) deoxyribonucleic acid (DNA) rose from 4919 to 119,125 copies/ml (Fig. 1a). Pembrolizumab, an anti-PD-1 inhibitor was considered owing to very limited further treatment options. Her archived neck lymph node specimens sent for PD-L1 expression with immunohistochemical staining revealed that the tumor proportion score was 100 and the combined positive score was 101, indicating that a promising response to ICI was expected. She denied any past medical history of tuberculosis. She then received pembrolizumab at 2 mg/kg every three weeks since September 2016. Her dyspnea and lung metastases improved and reduced in number dramatically after only two cycles of pembrolizumab, accompanied by a slump of EBV DNA to 83 copies/ml (Fig. 1b). She experienced mild irAEs with hypocortisolism and hypothyroidism which were effectively managed with hormone replacement therapy. In May 2018, she presented with a sudden onset of severe, colicky and localized right lower abdominal pain, projectile vomiting and bloody diarrhea with mucus, and persistent fever (temperature > 38.6 degrees Celsius). Initially immune-related enteritis/colitis was suspected and pembrolizumab was suspended. Positron emission tomography with integrated computed tomography (PET-CT) of the abdomen showed terminal ileitis with multiple enlarged mesenteric lymph nodes Colonoscopy was performed and the inflamed terminal ileum was biopsied, which exhibited multiple caseating granulomas with Langerhan cells, compatible with tuberculous ileitis (microscopic images captured by Nikon model DS-FI3 attached to Nikon Eclipse Ni microscope viewed by Application DS-L4 Viewer Software Ver1.2.0) (Fig. 2a). Otherwise, the pathology did not show any features of inflammatory bowel disease or immune-related enteritis. Though Ziehl–Neelsen stain did not identify any acid-fast bacilli, polymerase chain reaction test for TB of the ileal biopsy and interferon gamma release assay (IGRA) with QuantiFERON TB Gold Plus were both strongly positive. She immediately received anti-TB medication including rifampicin, ethambutol, pyrazinamide and isoniazid for 1 year following our microbiologist’s suggestion. Her terminal ileitis and the enlarged mesenteric lymphadenitis resolved in the follow-up PET-CT scan 9 months later. Colonoscopy performed 15 months after the last one confirmed complete resolution of her ileitis (Fig. 2b).

However, her lung metastases worsened again 7 months after anti-TB treatment (Fig. 1c), concurrent with an elevated plasma EBV DNA of 2826 copies/ml following pembrolizumab interruption. In light of her current progressive metastases and the prior extraordinary response to pembrolizumab, she was re-challenged with pembrolizumab in December 2018 together with her anti-TB treatment. PET-CT scan 5 months later showed promising tumour shrinkage (Fig. 1d). She is still receiving pembrolizumab with no evidence of TB relapse or other irAE.

## Discussion and conclusions

NPC, which is highly associated with EBV infection, is endemic in southern China including Hong Kong with an incidence of more than 20.0 per 100,000 [17]. Platinum-based chemotherapy is the standard first-line treatment for recurrent/metastatic NPC. Unfortunately, almost all patients relapse despite an initial response to chemotherapy. ICI alone, or in combination with systemic chemotherapy has established itself as an efficacious first- and second-line treatment in recurrent/metastatic head and neck squamous cell carcinoma. However, its role in recurrent/metastatic NPC is less defined. The phase I KEYNOTE-028 trial revealed an objective response of 26.3% with pembrolizumab [18]. Tumor response was highly correlated with PD-L1 expression in tumor cells.

Meanwhile, TB is also a notifiable and endemic disease in Hong Kong, with the notification rate of 54.0 per 100,000 population in 2019 [19], compared to 2.8 per 100,000 in the United States. It may remain latent for years or even decades. Containment of TB in its latent state is achieved through the surveillance of TB-directed CD4 + and CD8 + T cells [20], which happen to be the targets of ICIs. Indeed, the pathophysiology of TB reactivation following ICI therapy is complex and poorly understood, which involves both innate and acquired immune responses [15]. On one hand, exposure to TB leads to increased PD-1 and PD-L1 expression on natural killer (NK) cells of the innate system, resulting in release of interferon-γ (IFNγ) and cell lysis. Subsequent binding of PD-1 to PD-L1 and PD-L2 might inhibit further activation of NK cells, which prevents further tissue damage brought by inflammation. On the other hand, TB can also use the acquired immune response to avoid the host immune response by preventing IFNγ release and inhibiting CD8 + cell cytotoxicity via increased PD-1 expression. PD-1/PD-L1 inhibition by ICI, was shown in vitro to enhance CD8 + cell cytotoxicity against IFNγ-activated macrophages, leading to TB reactivation.

There have been some reports of TB reactivation following ICI therapy [3–15]. Intriguingly, patients in these reports who developed TB reactivation were those whose tumors also responded well to ICIs. It is postulated that TB reactivation which symbolizes an exaggerated immune response in the host might also predict a favorable tumor response to ICI therapy, though further studies are warranted to confirm such correlation.

A recent retrospective review using the US Food and Drug Administration Adverse Events Reporting System revealed that TB infection was only seen in 0.1% of patients after PD-1/PD-L1 therapy [16]. That said, the incidence would be much higher in TB-endemic regions. While international and local guidelines on screening TB before initiation of biological agents including anti-tumor necrosis factor-α (TNFα) for several chronic inflammatory diseases like rheumatoid arthritis have been published [21, 22], consensus on routine screening for TB before ICI therapy for cancer patients is still lacking. Chest radiograph and tuberculin test are recommended screening tools in these guidelines before anti-TNFα therapy. However, they may not be sensitive to diagnose acute/latent TB infection in cancer patients who are often immunocompromised and are also the high-risk groups more susceptible to TB reactivation after ICI therapy [23].

IGRA by detecting IFNγ released by T cells exposed to TB antigens, has been found more sensitive than the traditional methods to diagnose acute and latent TB, even in immunocompromised patients [24, 25]. New generations of IGRA were found even more sensitive than their earlier generations [24, 25]. Hopefully, international recommendations on TB screening with accurate and reliable methods before ICI initiation for cancer patients can be proposed and implemented routinely in TB-endemic regions.

In conclusion, we report the first gastrointestinal TB reactivation following ICI therapy for metastatic NPC. Accurate TB screening methods should be considered for latent TB infection before ICI therapy for cancer patients.

## Data Availability

The datasets analyzed during the current study are not publicly available due to patient privacy concerns but are available from the corresponding author on reasonable request.

## Abbreviations

DNA: Deoxyribonucleic acid
EBV: Epstein–Barr virus
ICI: Immune checkpoint inhibitors
IFNγ: Interferon-γ
IGRA: Interferon gamma release assay
irAE: Immune-related adverse events
NK: Natural killer
NPC: Nasopharyngeal carcinoma
PD-1: Programmed death-1
PD-L1: Programmed death-(ligand)1
PET-CT: Positron emission tomography with integrated computed tomography
TB: Tuberculosis
TNFα: Tumour necrosis factor-α
